# Approaches to Sex Education: Peer-Led or Teacher-Led?

**DOI:** 10.1371/journal.pmed.0050229

**Published:** 2008-11-25

**Authors:** David A Ross

## Abstract

David Ross discusses a new study of school-based peer-led sex education in London and whether it reduced unintended teenage pregnancy.

## Introduction

Prevention of early, unintended pregnancy, abortions, and sexually transmitted infections among adolescents is a very high priority in the United States and Europe, and the United Kingdom has a target to halve pregnancy rates among under-18-year-olds by 2010 [1]. School-based sexual health education provides an obvious approach, but evaluations of the effectiveness of such interventions, both within high-income [2,3] and low-income [4] countries have not been very encouraging. In this week's *PLoS Medicine*, Judith Stephenson and colleagues report the long-term results of the RIPPLE trial comparing peer-led and teacher-led approaches, which builds on previous studies of school-based sex education.

Most studies to date have depended upon self-reported behavioural outcomes such as pregnancy or abortion. Yet, self-reported sexual behaviour is notoriously prone to reporting errors, and there is considerable potential for biased misreporting after an intervention that aims to change behaviours. Also, most of these previous evaluations have only had relatively short follow-up, and many have used non-randomised designs.

Despite the relatively weak evidence of the effectiveness of sexual health education as a whole, except to improve knowledge, such education is widely implemented. This is justifiable on the grounds that providing young people with the knowledge and skills to improve their sexual health can be seen as a human right [5], and because strong evidence suggests sexual education does not encourage increased sexual activity or sexual risk [4].

**Linked Research Article**

This Perspective discusses the following new study published in *PLoS Medicine*:

Stephenson J, Strange V, Allen E, Copas A, Johnson A, et al. (2008) The long-term effects of a peer-led sex education programme (RIPPLE): A cluster randomised trial in schools in England. PLoS Med 5(11): e224. doi:10.1371/journal.pmed.0050224

Judith Stephenson and colleagues report on a cluster randomized trial in London of school-based peer-led sex education and whether it reduced unintended teenage pregnancy.

However, there is considerable dispute as to what the best strategy should be for sexual health education in school, with strong advocates for peer-led over the more standard teacher-led sex education [6].

## The RIPPLE Trial

The long-term evaluation of the effectiveness of peer-led sex education programmes in comparison with standard teacher-led sex education, studied in the RIPPLE trial, is therefore an important addition [7]. Twenty-seven secondary schools in England were randomly allocated to one of two groups: one group had older (16- to 17-year-old) peers lead three one-hour classes on topics such as sexual communication, condom use, contraception, and local sexual health services for 13- to 14-year-olds in their own school. The other group received the same number of sexual education classes but these were, as previously, led by teachers. The study showed that the peer-led programme was more popular with students [8] and the nature of the interaction in the peer-led sessions was different from the teacher-led sessions.

In a previous report, the RIPPLE researchers showed that at age 16 girls in the peer-led group reported fewer unintended pregnancies, although this difference was of borderline statistical significance (2.3% versus 3.3%, *p* = 0.07) [8]. In this week's *PLoS Medicine*, the researchers report the long-term results, with follow-up to age 20 years. Such long-term follow-up makes this study unique and important. But perhaps even more significantly, the researchers did not rely only upon self-reported data on pregnancies and abortions. They also identified all pregnancies and abortions among girls that were registered in routinely reported health service data through data linkage at the individual level. This is a major strength of the trial.

The results of the trial are, however, somewhat inconclusive. In terms of abortions, although there were fewer abortions reported by girls in the peer-education arm (weighted, adjusted odds ratio [OR] = 0.56; 95% confidence interval [CI] 0.31,1.02), this difference was not seen in the more objective outcome data on registered abortions from the data linkage study, either up to 18 years of age (OR = 1.19 [95% CI 0.81,1.75]), or up to 20 years of age (OR = 1.07 [95% CI 0.80,1.42]). In terms of pregnancies, although fewer pregnancies were self-reported by girls in the peer-education group (OR = 0.62 [95% CI 0.42,0.91]), the difference—though in the same direction—was not statistically significant in the analysis of the more objective data on registered live births either by 18.5 years of age (OR = 0.74 [95% CI 0.47,1.17]), or by 20.5 years of age (OR = 0.77 [95% CI 0.51,1.15]).

## The Importance and Implications of These Results for Research and Programmes

Despite the rather inconclusive findings, the long-term results of the RIPPLE trial are very important. First, they confirm the importance of including objective, biological outcomes in such trials, rather than only relying on self-reported data even of such salient events as pregnancy or abortion. Second, they give advocates of peer-led over teacher-led sex education reason to pause for thought. The peer-educator approach is far more labour intensive, requiring new cohorts of peer educators to be trained every year or two, and is often seen as more threatening than teacher-led sex education by school authorities. This might partly explain the very low uptake of schools participating in the trial, with less than 10% of eligible schools who were invited willing to participate, though apathy and the additional work related to the evaluation may also have been factors. Although the peer-led programmes were more popular with students, the borderline evidence of greater effectiveness in this trial should make education authorities think twice before replacing teacher-led sex education with peer-led, given the important financial and logistical barriers to large-scale adoption of peer-led sex education in schools.

Furthermore, there are many unanswered questions. Both the peer-led and the teacher-led sex education programmes were fairly minimal, at only three one-hour sessions. However, the SHARE trial in Scotland, which compared standard teacher-led sex education (seven to 12 sessions in total, largely devoted to provision of information and discussion) with a more intensive, specially designed teacher-led intervention (20 sessions in total across years 3 and 4 of secondary school [ages 13–15 years], with a focus on active learning and skills development), also found no impact on either reported or routinely registered pregnancy or abortion rates [9]. And perhaps such in-school interventions may need to be combined with interventions to change wider norms within society, including among parents.

## Looking Forward

Despite the inconclusive results of the RIPPLE trial, the scale and importance of immediate, short-term sexual and reproductive health problems among adolescents—and the potential for sex education during adolescence to influence adoption of norms and behaviours that could reap benefits throughout their subsequent adult lives [10]—means that we do not have the luxury of leaving things be. We must continue to develop and rigorously evaluate new approaches to reduce the adoption of sexual risk behaviours by young people. This is vital both in high-income countries such as the UK, and, even more importantly, in low-income countries, especially those with high maternal and infant mortality and incidence of HIV.

## Abbreviations

CI: confidence interval
OR: odds ratio
